# Weaker Self-Esteem in Adolescence Predicts Smoking

**DOI:** 10.1155/2015/687541

**Published:** 2015-07-26

**Authors:** Antti J. Saari, Jukka Kentala, Kari J. Mattila

**Affiliations:** ^1^University of Tampere, School of Medicine, 33014 Tampere, Finland; ^2^JYTA, Tunkkari Health Care Centre, Mäntöläntie 2, 69730 Veteli, Finland; ^3^Social and Health Services, P.O. Box 241, 65101 Vaasa, Finland; ^4^Centre of General Practice, Pirkanmaa Hospital District, P.O. Box 2000, 33521 Tampere, Finland

## Abstract

*Background*. To study whether weaker self-esteem in adolescence is connected with smoking behavior in adulthood. *Methods*. An age cohort born in 1979 responded to the Lawrence Self-Esteem Questionnaire (LAWSEQ) at the age of 16 (*n* = 1,072). Respondents' smoking behavior was monitored annually during adolescence and 75.3% (*n* = 813) of them remained nonsmokers during adolescence. A follow-up questionnaire eliciting smoking behavior was sent to the adolescent nonsmokers at the age of 29 years. Response rate at follow-up was 46.2% (*n* = 376). *Results*. Weaker self-esteem (LAWSEQ score ≥ 3) during the adolescence was not significantly associated with smoking in adulthood. However, those respondents who had weaker self-esteem in adolescence had increased risk of having been smoking regularly (adjusted OR 1.8, 95% CI 1.1–3.0) although not all of them were smokers at the time of the follow-up. *Conclusions*. Those with weaker self-esteem in adolescence are more likely to smoke regularly in adulthood.

## 1. Background

Tobacco smoking is a significant cause of a variety of problems for communities and individuals [1, 2]. WHO defines adolescents as people between 10 and 19 years old [3]. According to WHO, many adolescents are prone to develop unhealthy lifestyle and engage in risk behavior. Habitual smokers usually start smoking during adolescence [4, 5]. If smoking becomes a persistent habit, it greatly increases the risk of premature death [1].

A variety of personality-related factors have been shown to be associated with smoking. Problems with concentration had an independent effect on the probability of becoming a current smoker according to a Finnish twin study [6]. Among adults, personality factors such as neuroticism, poor self-discipline, impulsiveness, and low deliberation [7] as well as depressiveness [8] have been associated with smoking initiation and continuation. Continuing smoking and smoking cessation increase the risk of depression [9] and history of depression or anhedonia predicts smoking [10]. The link between smoking and depressive emotions seems to be due to problems with negative affect regulation; smoking is assumed to alleviate negative emotions [11]. There is no previously published study evaluating a potential association between self-esteem in adolescence and smoking in adulthood.

Weak self-esteem is a sign of vulnerability when it comes to affective disorders. According to Blatt and Zuroff's [12] theory of personality predispositions to depression, individuals with high levels of self-criticality and/or dependency are prone to develop depression after negative life-events. In addition to depressive symptoms, weak self-esteem is linked to weight problems [13] and social phobia [14]. Mental illness in young people predicts greater likelihood of starting smoking [15]. Decrease in self-esteem is linked with the development of social phobia and depression among adolescents [16].

A variety of methods to measure self-esteem have been developed. A commonly used method of measuring self-esteem is Lawrence Self-Esteem Questionnaire (LAWSEQ) [17], which has been shown to be a valid measure of an individual's self-esteem [18, 19].

In summary, smokers often have weaker self-esteem and those with weaker self-esteem are likely to smoke. The aim of this study was to find out if weaker self-esteem in adolescence is associated with smoking in adulthood. While the likelihood to start smoking cannot be measured, it is rational to try to find some measurable indicators that could be used to point out those individuals who are prone to start smoking.

## 2. Methods

The sample was picked from those age cohort subjects born in 1979 [20] who completed the Lawrence Self-Esteem Questionnaire (LAWSEQ) at the age of 16 while living in one of three Finnish towns and self-reported no smoking experimentations during ages of 12–16 (*n* = 813).

A back-translation (from Finnish into English) of our version of LAWSEQ is presented as an appendix. Two versions of LAWSEQ had been used in 1995, one in Finnish and one in Swedish. Those subjects whose mother tongue was Finnish had responded to the Finnish translation and vice versa. The responses to the LAWSEQ were used to assess respondents' self-esteem. We used a sum variable (later LAWSEQ score) that was the total number of points from the LAWSEQ questions. A “yes” answer to a question yielded 2 points, “cannot say” yielded 1 point, and “no” yielded 0 points except for the question “do you think that your parents usually like to hear about your own ideas?” where “yes” yielded 0 points, “cannot say” yielded 1 point, and “no” yielded 2 points. Consequently, LAWSEQ scores were between 0 (zero) and 20, where 0 represented the strongest and 20 the weakest self-esteem.

At the age of 16 the responses to LAWSEQ were received from 813 subjects. Of these subjects 51.7% (*n* = 402) got LAWSEQ score of 0, 1, or 2. Because of this we classified the respondents with LAWSEQ scores from 3 to 20 as having weaker self-esteem and those with LAWSEQ scores of 2 or less as having stronger self-esteem. Second, we classified the responses to separate self-esteem questions as weaker (1-2 points) and stronger (0 points). These responses were then used in binary logistic regression to see if individual responses representing weaker or stronger self-esteem had a connection with smoking behavior in adolescence or adulthood.

The subjects had also responded to a separate questionnaire about their smoking habits at the ages of 13, 14, 15, and 16. The question we used to classify respondents into adolescent nonsmokers (and study population) or adolescent smokers (and exclusion) was “do you smoke?” (no/yes). Their parents' smoking behavior was also elicited annually in the questionnaire. The respondent's gender and parents' smoking behavior were noted as potential confounding factors for smoking in adolescence.

In 2008 we mailed a follow-up questionnaire to assess the smoking of the cohort in adulthood. The addresses of the sample population were obtained from the Finnish Population Register Centre. We sent the follow-up questionnaires in 2008. The response rate was 46.2% (*n* = 376).

We used two methods for measuring smoking behavior from the responses to the follow-up questionnaire. Those respondents answering “yes” to the question “do you smoke?” were classified as being smokers in adulthood. Those respondents answering “yes” to both questions “during your life have you smoked more than 5 packs of cigarettes or cigars or smoked at least an estimated equivalent amount of loose or pipe tobacco?” (yes/no) and “do you smoke or have you smoked tobacco products regularly, in other words daily or nearly daily?” (yes/no) were classified as having been smoking regularly. The respondent's gender was noted as a potential confounding factor for being a smoker in adulthood or having been smoking regularly.

In the follow-up envelopes there was also a cover letter describing the purpose and methodology of the study and enclosing a consent form. Only questionnaires returned with a signed consent form were used as data. The Ethics Committee of the Pirkanmaa Hospital District, Finland, approved the study protocol (R08017).

We used IBM SPSS 20.0 for the statistical analyses. Nonrespondents were excluded from the analysis. The data was analyzed using frequencies, percentages, cross-tabulation, and Fisher's exact test. Independent samples *t*-test was used to analyze differences in LAWSEQ scores between smokers and nonsmokers. Logistic regression analysis was performed to obtain odds ratios (OR) and 95% confidence intervals (CI). The dependent variable was smoking in adulthood or having been smoking regularly. Multivariate analyses were also conducted to adjust for confounders.

## 3. Results

Of all the respondents, 8.8% (*n* = 33) were adulthood smokers. Among the respondents, 7.6% (*n* = 18) females and 11.0% (*n* = 15) males were smokers. There were no statistically significant gender differences in smoking.

**Figure 1:**
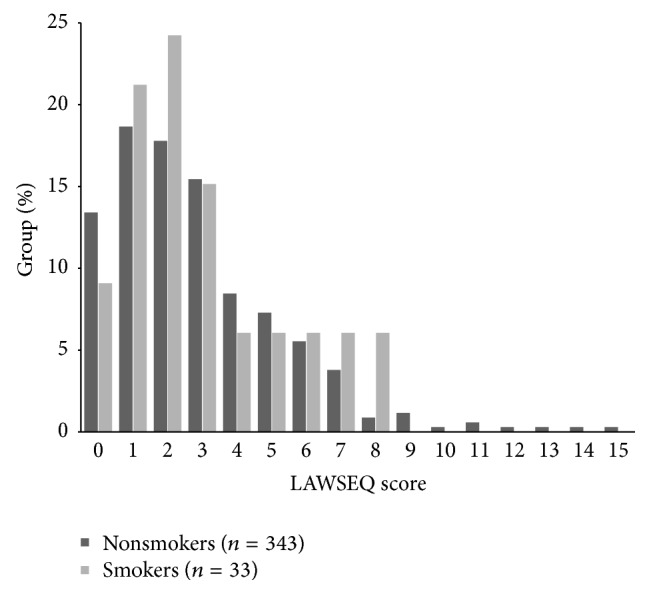
Distribution of LAWSEQ score measured at the age of 16 in the groups of adult smokers and adult nonsmokers. Higher scores indicate weaker self-esteem.

Median LAWSEQ score in the study population was 3. Of all the respondents, 76.1% (*n* = 286) scored less than the median value 3 (three). This was considered to be close enough to the original distribution of LAWSEQ scores (see Section 2) and thus the cut-point of median was accepted for dichotomization of LAWSEQ scores to those representing stronger or weaker self-esteem. There were no statistically significant differences in LAWSEQ scores between the groups of adulthood smokers and adulthood nonsmokers (Figure 1). Weaker self-esteem during the adolescence was not significantly associated with smoking in adulthood (Table 1).

Among all respondents, 24.5% (*n* = 58) females and 36.7% (*n* = 51) males had been smoking regularly (*p* = 0.014). Those with weaker self-esteem during adolescence were more likely to have been smoking regularly (adjusted OR 1.8, 95% CI 1.1–3.0) (Table 1).

When looking at each LAWSEQ question separately, responses to the separate LAWSEQ questions were not associated with increased or decreased risk of being a smoker in adulthood.

## 4. Conclusions

Smoking behavior in adulthood seems to be connected with higher LAWSEQ score and thus lower self-esteem in adolescence. It seems that the effect of poor self-esteem does not affect adolescents' smoking during adolescence, but as time passes and they grow into adulthood, poor self-esteem has a predictive effect on their smoking behavior. The back-translation of LAWSEQ was partially noncomparable (see appendix) and this may have caused bias on the results. The reliability of LAWSEQ scores has been found satisfactory in recent analyses [21]. However, LAWSEQ has not been validated in Finnish populations and cultural differences may also have an effect on the adequacy of the translation used in this study. It should also be kept in mind that measuring self-esteem is controversial. It is not possible to determine one value where LAWSEQ score interpretation changes from strong self-esteem to weak self-esteem. In this study we used the LAWSEQ score cut-point where half of the originally tested subjects scored under and another half over the cut-point value. Our justification on this method is its simplicity; tests to define personality-related factors should be compared to the population to which the test has been conducted.

Our respondents mostly had high education, were living in a pair relationship, and perceived their health to be very good; thus according to existing knowledge they are unlikely to be smokers. If we had been able to analyze the adulthood smoking behavior of the nonrespondents, it is possible that there would have been more of those with problems with both self-esteem and smoking. This selection bias is likely to undermine our results. Half of the cohort received up to four brief tobacco interventions in school age. This is unlikely to cause any bias in our study since the intervention did not prove effective in long-term follow-up [22]. Recent evidence of the long-term ineffectiveness of cessation interventions concurs with the assumption that earlier interventions did not bias our results [23].

Since the findings reported here have not been observed before, we call for further studies to elucidate further the relationship between self-esteem and smoking behavior. Using a different method for grading self-esteem and/or collecting the responses at a different age or from a different population could have been useful. SES (Rosenberg Self-esteem Scale) [24] has been used as a golden standard to measure self-esteem. It has been validated in many different countries and translated into many languages [25, 26]. The Finnish translation of SES has been used in a study concluding that self-esteem is affected by environmental factors [27]. However, SES had not been translated to Finnish at 1996 when this study was put into practice.

In conclusion weaker self-esteem in adolescence is associated with smoking in adulthood. Problems with self-esteem may be a practical indicator of a specific need for antismoking interventions, and we call for further studies to see if adolescents with self-esteem issues benefit from antismoking interventions.

## Figures and Tables

**Table 1:** Odds for being smoker in adulthood or having been smoking regularly among the groups with weaker or stronger self-esteem.

	Smoking behavior			
	One variable		Adjusted^*∗*^	
	OR (95% CI)	*p* value	OR (95% CI)	*p* value
	Smoker in adulthood			
Self-esteem		0.370		0.414
Stronger	1		1	
Weaker	1.43 (0.65–3.13)		1.39 (0.63–3.05)	

	Has been smoking regularly			
Self-esteem		**0.018**		**0.030**
Stronger	1		1	
Weaker	1.82 (1.11–3.01)		1.75 (1.05–2.91)	

OR = odds ratio, CI = confidence interval, stronger = LAWSEQ score <3, and weaker = LAWSEQ score 3 or more.

^*∗*^The adjusted model includes gender and smoking of parents.

## Appendix

1. Are there many things you would like to change about yourself?
2. Do you think your school friends often talk ill of you?
3. Do others often think you are lying?
4. Do your parents usually want to hear your thoughts?
5. Do you usually feel yourself stupid when talking with your parents?
6. Do you often find it difficult to address your teacher?
7. If there is something that you need to tell your teacher, do you usually feel yourself stupid?
8. Do other pupils often run into conflicts with you?
9. Do you often feel yourself lonely at school?
10. Do you often need to find new friends because your old friends are with someone else?
